# The 3-dimensional printing for dental tissue regeneration: the state of the art and future challenges

**DOI:** 10.3389/fbioe.2024.1356580

**Published:** 2024-02-22

**Authors:** Fengxiao Zhao, Zhijun Zhang, Weihua Guo

**Affiliations:** ^1^ State Key Laboratory of Oral Diseases, West China Hospital of Stomatology, Sichuan University, Chengdu, China; ^2^ National Engineering Laboratory for Oral Regenerative Medicine, West China Hospital of Stomatology, Sichuan University, Chengdu, China; ^3^ Department of Pediatric Dentistry, West China School of Stomatology, Sichuan University, Chengdu, China; ^4^ Yunnan Key Laboratory of Stomatology, The Affiliated Hospital of Stomatology, School of Stomatology, Kunming Medical University, Kunming, China

**Keywords:** 3D printing, regenerative dentistry, bioprinting, biomaterial, dentin-pulp complex, periodontal regeneration, tissue engineering

## Abstract

Tooth loss or damage poses great threaten to oral and general health. While contemporary clinical treatments have enabled tooth restoration to a certain extent, achieving functional tooth regeneration remains a challenging task due to the intricate and hierarchically organized architecture of teeth. The past few decades have seen a rapid development of three-dimensional (3D) printing technology, which has provided new breakthroughs in the field of tissue engineering and regenerative dentistry. This review outlined the bioactive materials and stem/progenitor cells used in dental regeneration, summarized recent advancements in the application of 3D printing technology for tooth and tooth-supporting tissue regeneration, including dental pulp, dentin, periodontal ligament, alveolar bone and so on. It also discussed current obstacles and potential future directions, aiming to inspire innovative ideas and encourage further development in regenerative medicine.

## 1 Introduction

As an important craniofacial organ, teeth perform crucial functions including mastication and pronunciation. Loss or damaged tooth can be caused by trauma, bacterial infection, gene disease, or craniofacial cancer, posing great threaten to oral health even general health. Though there are already available treatments to remove damaged tissue and restore morphology and function of tooth to a certain extent, such as resin restoration for caries, root canal therapy for infected pulp, dental implants for edentulism, all of these clinical procedures have limited prognosis and fail to achieve full replacement of native tissue. Therefore, regenerative therapeutic strategies are urgently needed as alternative methods to create dental tissue substitutes with original structure and function (Cooper, 2009; Sallum et al., 2019; Siddiqui et al., 2022).

The natural tooth features a sophisticated and hierarchically organized architecture. Given that, the reproduction of complicated structure and integrated complex tissue remain challenging (Duailibi et al., 2006; Yildirim et al., 2011). Nevertheless, the advancement of biomaterials, further exploration of stem/progenitor cells, as well as innovative biofabrication technologies are believed to facilitate dental tissue functional regeneration.

Currently, three-dimensional (3D) printing technology, or additive manufacturing, have developed tremendously, providing new breakthrough for tissue/organ regeneration (Mota et al., 2020). As a rapid prototyping technology, 3D printing precisely deposit cells, materials, and bioactive agents into pre-defined locations in a “layer-by-layer” manner and construct intricated biomimetic structures (Murphy and Atala, 2014). Compared with conventional methods of tissue engineering, a 3D-printed scaffold holds many advantages, such like high fidelity, customized topographies, and superior production efficiency (Zhou et al., 2020; Vu et al., 2021). Up to now, numerous studies have focused on the application of 3D printing strategy in the field of regenerative dentistry (Ma et al., 2019).

**FIGURE 1:**
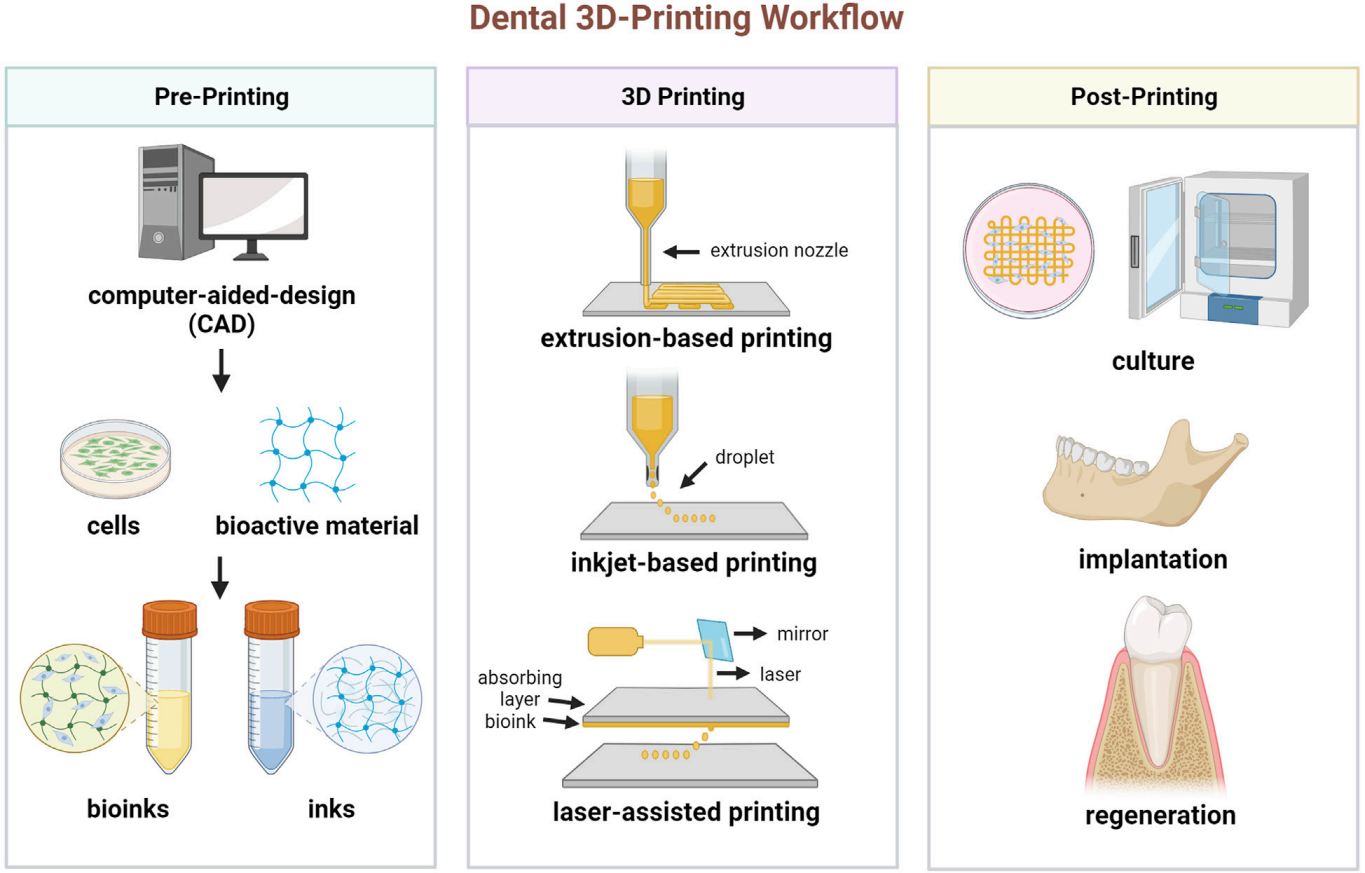
Schematic diagram of the process for 3D printing dental tissue. (Created with BioRender.com).

The process workflow of 3D printing dental tissue is briefly illustrated in Figure 1. The first step is to create a 3D model containing the macroscopic and microscopic structure of the scaffold. The data for the model can be acquired by computed tomography (CT) scanning for post-processing or directly by computer design, also known as computer-aided-design (CAD). Based on the target tissue, suitable biomaterials and cells are selected and prepared, as well as other bioactive additions. Then, the developed inks (only biomaterial) or bioinks (incorporating with living cells) are loaded into the 3D printer. The common 3D printing techniques include extrusion-based printing, as well as several variants such like fused deposition modeling (FDM)and melt electrowritten (MEW), inkjet-based printing and laser-assisted printing (e.g., stereo lithography appearance, SLA; digital light processing, DLP, selective laser sintering, SLS) (Mota et al., 2020). These techniques have variable features, which should be taken into account for manufacturing (Table 1). For cell-free printing, cell seeding is commonly performed after scaffold fabrication has been completed. Eventually, 3D printed constructs are cultured *in vitro* or implanted *in vivo* for biological regeneration.

**TABLE 1:** Techniques used in 3D printing of dental tissue.

Technique	Advantages	Disadvantages	Application	Reference
Extrusion-based printing	• Extensive sources of material	• Limited printing resolution	Dentin-pulp complex, alveolar bone, PDL, cementum, whole tooth, bio-root	Gao et al. (2019)
	• Low cost	• Slow printing speed		
		• Shearing force unfavorable to cells		
Inkjet-based printing	• Relatively fast printing speed	• viscosity limitations of ink	Dental pulp, alveolar bone	Li et al. (2020a)
	• High resolution			
	• High cell viability			
Laser-assisted printing	• High printing speed	• Expensive cost	Dental pulp, bio-root, periodontal tissue, alveolar bone, PDL, cementum	Quan et al. (2020)
	• High precision	• Only light-curing materials		

Even if 3D bioprinting still faces some hurdles, it has become an attractive and promising option to tooth and tooth-supporting tissue regeneration. In this review, we summarized the bioactive materials, cells, and recent progresses in the field of 3D printing for dental tissue, and then, we highlighted the challenges that have appeared in the tooth regeneration, and envision future directions for regenerative dentistry.

## 2 Biomaterials in 3D printing for dental tissue regeneration

Several types of bioactive material have been used to manufacture 3D-printed constructs for dental tissue regeneration, mainly including hydrogels, bioceramics, synthetic polymers and decellularized extracellular matrix (dECM). For mimicking the natural tissue microenvironment, hybrid bioactive ink or bioink are also developed (Table 2).

**TABLE 2:** Biomaterials used for dental tissue regeneration and their effects.

Type	Material	Bioink formulation	Technique	Effects	Reference
Natural hydrogel	Collagen	0.2% collagen type I, 0.5% agarose	Inkjet	Form vascular networks with hDPSCs and HUVECs *ex vivo*	Duarte Campos et al. (2020)
		4% collagen	Extrusion	Form aligned PDL fibers *in vivo*	Lee et al. (2021b)
	Chitosan	Chitosan, sodium alginate, carbon nanotube	Extrusion	Antimicrobial effect on P. gingivalis; promote the proliferation of hPDLCs	Suo et al. (2023)
		Chitosan	Extrusion	Promote cell adhesion and viability of hDPSCs	EzEldeen et al. (2021)
	Alginate	4% alginate, 20% gelatin	Extrusion	Promote cell adhesion, proliferation and osteogenic/odontoblastic differentiation of hDPSCs	Yu et al. (2019)
	β-TCP	β-TCP/PLGA at 75:25 ratio	Extrusion	Promote cell adhesion, proliferation and osteogenic differentiation of hDPSCs	Cao et al. (2020)
		β-TCP, TPP, CMC	Extrusion	Promote osteoblastic differentiation of DPSCs	Fahimipour et al. (2018)
	MTA	0.5g MTA, 6 g PCL	Inkjet	Promote cell adhesion and growth of DPSCs	Wu et al. (2016)
	Calcium silicate	CS, PCL	FDM	Induce odontogenic differentiation of hDPSCs	Huang et al. (2018)
		Strontium-doped CS, PCL	Extrusion	Enhance bone regeneration *in vivo*	Wang et al. (2021)
	Hydroxyapatite	Nano-HA, sodium alginate, gelatin	Extrusion	Promote cell survival, proliferation and osteogenic differentiation of hPDLSCs	Tian et al. (2021)
GelMA	GelMA	GelMA conjugated with BMP-peptide	Extrusion	Promote odontogenic differentiation of hDPSCs	Park et al. (2020)
		GelMA, CS	Extrusion	Promote odontogenic differentiation of hDPSCs	Choi et al. (2022)
		15% GelMA, 10% CS	Extrusion	Promote odontogenic differentiation of hDPSCs	Lin et al. (2021b)
		10% GelMA, bioglass nanoparticles	Extrusion	Promote osteogenic/cementogenic differentiation of hPDLCs	Mei et al. (2022)
Synthetic polymer	PLA	PLA	FDM	Promote odontogenic differentiation of DPSCs	Feng et al. (2021)
	PCL	PCL, reduced graphene oxide	Extrusion	Promote cell adhesion of DPSCs	Park et al. (2021)
	wPU	wPU, boric acid	FDM	Promote osteogenic differentiation of DPSCs	Hung et al. (2016)
dECM	dECM	DFCs-derived dECM, GelMA	DLP, DIW	regenerate periodontium with hDFSCs *in vivo*	Yang et al. (2023)
		Bone-derived dECM, β-TCP	Extrusion	Promote the osteo/odontogenic differentiation of hDPSCs	Kim et al. (2022)
	TDM	TDM, 30% PCL	Extrusion	Promote cell adhesion, proliferation and odontogenic differentiation of DFSCs	Huang et al. (2023)

### 2.1 Natural hydrogel

#### 2.1.1 Collagen

As the most abundant component in extracellular matrices (ECM), collagen is widely used as the scaffold material in tissue engineering (Wang Y. et al., 2023). Collagen contains arginine-glycine-aspartic acid (RGD)-motifs, which capable of mediate cell adhesion (Wu et al., 2021). Collagen is not only the main component of dental pulp and periodontium, but also the biomineralization matrix of dentin and bone. Thus, collagen-based bioink have been used for the reconstruction of dental pulp, periodontal ligament, and alveolar bone (Duarte Campos et al., 2020; Lee U.-L. et al., 2021; Li et al., 2022). Despite good biocompatibility, flexibility, and low immunogenicity, the poor mechanical property limit collagen’s use as bioinks. Incorporation with other hydrogel or organic/inorganic materials may be a feasible approach to improve mechanical stability and broaden the application boundaries of collagen-based bioink (Lee J. M. et al., 2021).

#### 2.1.2 Chitosan

Derived from exoskeletons and shells of crustaceans, chitosan is abundant in nature and known as a widely used scaffold material in tissue engineering. Chitosan possesses high biocompatibility, hydrophilicity, and controllable biodegradability (Szulc and Lewandowska, 2022). Moreover, a broad spectrum of antibacterial properties makes it unique advantage for the dental application (Li Y. et al., 2020). Numerous studies have proven that 3D-printed chitosan-based scaffold can be employed for dental tissue regeneration due to its tunable physiochemical properties and favorable biological response (Suo et al., 2023). EzEldeen et al. (2021) explored the effects of chitosan source (animal vs. fungal), co-polymerization with gelatine, and crosslinking agent (3-glycidyloxyproply trimethoxysilane, GPTMS or genipin) on scaffold properties and biological response. However, the low mechanical property and sterilization strict the application of chitosan-based hydrogel in the bioprinting field (Lazaridou et al., 2022). Therefore, many attempts have been made to enhance the mechanical strength of chitosan by functional groups modification and co-crosslinking with other molecules (Liu et al., 2021; Salar Amoli et al., 2022).

#### 2.1.3 Alginate

As a natural biopolymer, alginate attracted scientists’ focus for its water solubility and rapidly cross-linked by calcium ions at room temperature. Besides, alginate possesses proper mechanical property, tunable viscosity and acceptable printability, which make it suitable for bioprinting technique (Gao et al., 2021). However, the pure alginate lacks RGD motifs for cell attachment, and the enzyme that cleave alginate chain is absent in human body, leading to the uncontrollable biodegradation *in vivo*. Given that, alginate is often used with other materials to form composites for bioinks (Rastogi and Kandasubramanian, 2019; Yu et al., 2019).

#### 2.1.4 Bioceramics

Bioceramic materials, including β-tricalcium phosphate (β-TCP), hydroxyapatite (HA), bioglass, calcium silicate (CS) and so on, have been well investigated as scaffold materials in traditional tissue engineering manufacturing for their excellent biocompatibility, favorable bioactivity and easy tailored characters (Baino et al., 2015). Owing to the intrinsic similar to the inorganic composition of bone and tooth, bioceramics are often used for the regeneration of hard tissues. But they also have disadvantages of low fracture resistance, low flexural resistance and wettability (Raveau and Jordana, 2020).

Typically, bioceramics often added as fillers to the polymer matrix to obtain ideal physicochemical and mechanical properties. A study conducted by Ahlfeld et al. (2023) indicated that varieties of mineral fillers, including CaCO_3_, SrCO_3_, strontium-modified hydroxyapatite (SrHAp) or tricalcium phosphates, integrated into a PLGA matrix can remarkably modify the degradation behavior of printed scaffold and an increase of the specific alkaline phosphatase activity were observed. Besides, mesoporous calcium silicate is also used in hard tissue regeneration as a component of biomaterial inks because of its ability to sustained release Si ions and other bioactive agents (Huang et al., 2018; Yeh et al., 2022). A bioactive strontium-doped calcium silicate (SrCS) scaffold released Sr and Si ions even after 6 months and enhanced secretion of osteogenic-related proteins (Wang et al., 2021).

Commonly, hydrogels are mixed with bioceramics particles for enhanced mechanical stability and adjustable biological properties. Tian et al. explored a novel bioink composed of sodium alginate (SA), gelatin (Gel), and nano-hydroxyapatite (na-HA). The SA/Gel/na-HA hydrogel exhibited shear-thinning behavior, optimized equilibrium swelling rate and compression modulus, as well as significantly improved the osteogenic differentiation of human periodontal ligament stem cells (hDPLSCs) (Tian et al., 2021).

#### 2.1.5 GelMA

Gelatin methacryloyl (GelMA) is a gelatin derivative modified by methacrylamide and methacrylate groups. Resembling the native ECM, GelMA contains many RGD-sequences for cell attachment as well as matrix metalloproteinase (MMP)- degradable motifs for cell remolding (Yue et al., 2015). As a photo-crosslinked hydrogel, GelMA undergoes photoinitiated radical polymerization without chemical crosslinkers that may be detrimental to cells (Klotz et al., 2016). For its tunable mechanical characteristics and suitable biological properties, GelMA is a versatile matrix that can be vastly used in biomedical application (Sakr et al., 2022). Numerous studies have proven that GelMA-based scaffolds can promote cell viability and osteo/odontogenetic differentiation of dental stem cells, including DPSCs, PDLSCs and SCAPs (Ha et al., 2020; Buyuksungur et al., 2021; Zhu et al., 2023).

Not only served as cell delivery vehicles in bioprinting, GelMA also plays a key role in drug and growth factor delivery due to its biodegradable properties. Park et al. (2020) developed bone morphogenetic protein (BMP) peptide-tethering bioink by conjugating peptides to GelMA. The results showed that BMP-mimicking peptide remained in bioprinted construct for more than 50% after 3 weeks and robustly increased the expression of odontogenic-related genes of hDPSCs.

A study conducted by Choi et al. (2022) used two different types of MTA (ProRoot MTA and Endosem Zr) combined with GelMA and evaluated the biological effects on hDPSCs. Although 3D-printed MTA/GelMA scaffolds showed more calcium deposition, elevated expression of odontogenetic-related genes was not statistically significant. Lin et al. fabricated the composite scaffold with CS reinforced-GelMA bioink. The CS/GelMA bioink is capable of inducing odontogenic differentiation of hDPSCs. Another study showed that the addition of mesoporous bioactive glass nanoparticles significantly enhanced shape fidelity, surface roughness, and bioactivity (Mei et al., 2022).

#### 2.1.6 Synthetic polymer

Synthetic polymers are another kind of vastly used materials in tissue engineering. They have the advantages of adjustable physical and chemical properties, which makes it suitable candidates for the dental tissue regeneration.

Polylactic acid (PLA) and poly(L-lactic-co-glycolic) acid (PLGA) are well-established polymers for their biosecurity approved by Food and Drug Administration (FDA) and excellent manufacturing ability. Moreover, PLGA possesses tunable degradation rate by different the ratio of lactic acid to glycolic acid, which can be employed as carries of biomedical substance (Danhier et al., 2012). However, both PLA and PLGA undergo hydrolytic degradation and generates acid degradation products that may trigger local inflammation *in vivo*, thus confine their application (Ramot et al., 2016; Li et al., 2017). Polycaprolactone (PCL) is also conventional scaffold material routinely used in 3D-printing and has desired biocompatibility and biodegradability. Nevertheless, the lack of cell-anchoring site and hydrophobic surface lead to the low bioactivity of synthetic polymers (Place et al., 2009). Various attempts have been made for the optimization of biological properties, including surface modification, biomaterial coating, introduction of functional groups and so on (Bow et al., 2021; Feng et al., 2021; Park et al., 2021).

Given that these traditional polymers often require organic solvent for dissolving in 3D printing process and cause solvent residue in the final construct, in recent years, waterborne biodegradable polyurethane (wPU) have shown great potential as 3D-printing materials (Hung et al., 2016). However, very few studies are found about PU application in the dental tissue regeneration. A study published recently employed PU scaffolds coated with boric acid. The wPU/boric acid scaffold can trigger the osteogenic differentiation of DPSCs without significantly reducing cell viability and proliferation (Çelebi-Saltik et al., 2023).

#### 2.1.7 Decellularized extracellular matrix

dECM refers to the natural tissue with the removal of immunogenic cellular components while three-dimensional network of ECM and biological molecules were remained, including various proteins and polysaccharides. Compared with other hydrogels, dECM provides not only mechanical support and adhesion cites for cells, but also biological cues to impact cell behavior by cell-ECM interactions (Zhang et al., 2022).

Several studies have proven that dECM derived from dental tissue/cells hold the capacities of recruiting stem cells, promoting osteogenesis, and forming pulp-like tissue (Smith et al., 2012; Heng et al., 2016; Nowwarote et al., 2021; Tan et al., 2021). Due to the rising demand for biomimetic microenvironments of 3D printed constructs, dECM is gradually becoming a promising bioink material (Kim et al., 2022; Yang et al., 2023).

Treated dentin matrix (TDM), also termed as “demineralized dentin matrix” (DDM), is a kind of specific dECM that referred to dentin matrix after a certain degree of demineralization treatment. With exposed dentin tubules and collagen fibrils, TDM released abundant dentinogenetic-related biomolecules including COL-1, DSPP, DMP-1, TGF-β1, decorin, biglycan, and created an inductive microenvironment for dentin regeneration (Guo et al., 2009; Li et al., 2011). Athirasala et al. (2018) mixed the insoluble components of the DDM with alginate in 1:1 ratio, and then added extracted soluble dentin molecules, dramatically promoting hydrogel-encapsuled SCAPs differentiate into dentin. Besides, TDM particles are also used to form particle-based bioink and regenerate specific dental tissue or whole tooth (Huang et al., 2023).

## 3 The stem/progenitor cells used in the dentistry 3D printing

### 3.1 Dental pulp stem cells (DPSCs)

**FIGURE 2:**
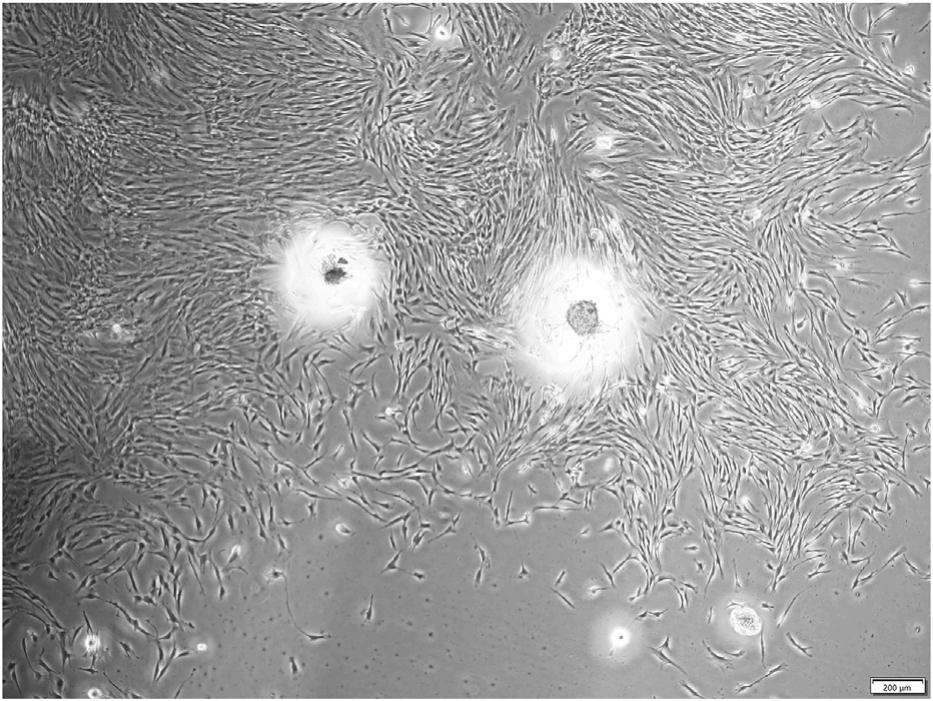
Primary of human dental pulp stem cells (hDPSCs).

Dental pulp stem cells (DPSCs) was firstly isolated and identified by Shi and Gronthos in 2000 (Gronthos et al., 2000). As the mesenchymal stem cell, DPSCs possess self-renewal potential and multi-directional differentiation capacity, being capable of differentiating into odontoblasts, chondrocytes, adipocytes, and neural-like cells *in vitro* and regenerate dentin-pulp complex *in vivo* (Gronthos et al., 2002; Wei et al., 2007). The typical microscopic morphology of human DPSCs is shown in Figure 2. Compared with bone marrow-derived mesenchymal stem cells (BMMSCs), DPSCs express more odontoblast-related genes, such as ALP, DSPP, and DMP-1 (Yamada et al., 2006). Due to their wide sources and minimally invasive process (Ferrúa et al., 2017), DPSCs have already became a kind of ideal seeding cells for tooth tissue engineering (Anitua et al., 2018; Liu P. et al., 2022). DPSCs loaded 3D-printed PLA/HA scaffold exhibited higher degree of mineralization than the cell-free scaffold (Chen et al., 2021). Some studies have also used gene-modified dental pulp stem cells for 3D printing (Wang W. et al., 2023).

### 3.2 Stem cells from human exfoliated deciduous teeth (SHEDs)

In 2003, Miura firstly found that exfoliated human deciduous tooth contains a population of multipotent stem cells and named it stem cells from human exfoliated deciduous teeth (SHED) (Miura et al., 2003). Similar to DPSC, SHEDs are capable of differentiating into odontoblast, adipocytes and neural-like cells. Owing to the immaturity of SHEDs, several studies suggested that SHEDs are more proliferative than DPSCs and BMMSCs (Nakamura et al., 2009; Wang et al., 2012). In addition to the high proliferation rate and multilineage differentiation potential, the noninvasive harvesting procedure also make SHEDs a promising cell source for the regeneration of tooth, cartilage and neural tissue (Yang X. et al., 2019; Pereira et al., 2019; Mahdavi-Jouibari et al., 2023).

### 3.3 Periodontal ligament stem cells (PDLSCs)

Periodontal ligament stem cells (PDLSCs), firstly isolated from periodontal ligament by Seo in 2004, are progenitor cells of cementoblasts, periodontal ligament fibroblasts and alveolar bone osteoblasts. Seo also found that PDLSCs hold the potential to generate cementum/PDL-like structure in immunocompromised mice (Seo et al., 2004). As one of the well-established stem cells in the field of regenerative dentistry, PDLSCs have been considered as ideal seeding cells of 3D printed scaffold that are capable of regenerating periodontal tissue both *in vivo* and *in vitro* (Park et al., 2012; Lee et al., 2014; Daghrery et al., 2023).

### 3.4 Stem cells from apical papilla (SCAPs)

The stem cells from apical papilla (SCAPs), located on the exterior of the immature permanent tooth root foramen area, plays an important role in the root formation and development. In 2006, Sonoma collected human root apical papillae from young adults (18–20 years old) extracted third molars and isolated stem/progenitor cells that express the mesenchymal stem cells marker: STRO-1 (Sonoyama et al., 2006). It is demonstrated that SCAPs hold the capacity to regenerate dentin-like and pulp-like tissue *in vivo* (Sonoyama et al., 2006; Huang et al., 2010; Hilkens et al., 2017). Besides, SCAPs can also differentiate into cementoblasts *in vitro*, which hold the promise for periodontal regeneration (Ebadi et al., 2023).

### 3.5 Dental follicle stem cells (DFSCs)

The dental follicle is an ectomesenchyme-derived connective tissue that surrounds the enamel organ and dental papilla, playing a key role in periodontal tissue formation and tooth eruption (Zhang et al., 2019). Dental follicle stem cells (DFSCs) are mesenchymal stem cells isolated from dental follicle tissue and are considered as precursor cells of cementoblasts, osteoblasts and periodontal ligament cells (Morsczeck et al., 2005). The present researches have confirmed that DFSCs hold the capacity of regenerating bone, dentin, periodontal tissue and tooth root-like tissue *in vivo* (Guo et al., 2009; Guo et al., 2012; Yang H. et al., 2019; Meng et al., 2022). Though limited literatures reported the application of DFSCs in dental tissue bioprinting, it is gradually attracting more and more attention.

### 3.6 Others

In addition to the cells mentioned above, there also various kinds of stem/progenitor cells used for tooth and periodontal regeneration, such like bone marrow derived mesenchymal stem cells (BMSCs) (Golafshan et al., 2023; Miao et al., 2023), Hertwig’s epithelial root sheath (HERS) cells (Tang et al., 2022), dental papilla cells (DPCs), gingival fibroblast cells (GFs) (Wang et al., 2021; Liu et al., 2023) and induced pluripotent stem cells (iPSCs) (Kim et al., 2023).

## 4 3D printing for dental tissue regeneration

The natural tooth is composed of both hard tissue (dental enamel, dentin, cementum and alveolar bone) and soft tissue (dental pulp, periodontal ligament, gingiva). Tables 3, 4 summarized recent advancements in dental soft and hard tissue printing respectively. Notably, natural tissues and organs typically exhibit compartmentalized architecture with an intrinsic integration of diverse components, and the tooth follows suit. Therefore, Table 5 presents the applications of 3D printing/bioprinting in the regeneration of composite tissue, including the dentin-pulp complex, periodontal complex, as well as whole tooth and tooth root.

**TABLE 3:** Applications of dental soft tissue printing.

Tissue	Bioink	Cells/biological supplement	Technique	Results	Reference
Dental pulp	Collagen, agarose	DPSCs^a^, HUVECs^a^	Inkjet	Successful vasculogenesis in root canal *in vitro*	Duarte Campos et al. (2020)
Dental pulp	GelMA microspheres	hDPSCs	DLP	Regenerate full-length dental pulp with blood vessels and nerve in minipigs model	Qian et al. (2023)
PDL	Collagen	PDLSCs	Extrusion	Fabricate waveform microfibers under shear stress	Lin et al. (2021a)
PDL	Collagen	hPDLSCs^a^/FGF-2	Extrusion	Produce connective tissue between titanium implant and bone	Lee et al. (2021b)
PDL	(wax mold)	PDLSCs	Extrusion	Form organized PDL cells	Kim and Park (2020)
Gingiva	Alginate, gelatin	GFs^a^/i-PRF	Extrusion	Regenerate oral soft tissue and enhance angiogenic activity *in vivo*	Yi et al. (2022)
Gingiva	Acellular dermal matrix, gelatin, sodium alginate	GFs^a^	Extrusion	Increase the amount of keratinized gingiva *in vivo*	Liu et al. (2023)

^a^ Cells composing bioinks.

**TABLE 4:** Applications of dental hard tissue printing.

Tissue	Bioink	Cells/biological supplement	Technique	Results	Reference
Enamel	HAp nanorods; resin	—	Extrusion	Multi-scale ordered tooth crown	Zhao et al. (2022)
Enamel	Carboxymethyl chitosan, alginate	HAT-7 cells	Extrusion	Promote ameloblast differentiation and matrix mineralization *in vitro*	Mohabatpour et al. (2022)
Dentin	Biodentine, PCL	hDPSCs	Extrusion	Apatite formation; promote odontogenic proliferation	Ho et al. (2018)
Dentin	Calcium silicate/calcium sulfate, PCL	hDPSCs/quercetin	Extrusion	Promote odontogenic differentiation	Yeh et al. (2022)
Dentin	Fibrinogen, gelatin, DDM particle	DPSCs^a^	Extrusion	Tooth-shaped dental construct *in vitro*	Han et al. (2021)
Dentin	PLA	DPSCs/titania coating	FDM	Templated biomineralization and odontogenic differentiation of DPSCs	Feng et al. (2021)
Dentin	Bone-derived dECM, β-TCP	DPSCs^a^	Extrusion	Promote the osteo/odontogenic differentiation	Kim et al. (2022)
Cementum	PCL	PDLSCs, growth factors	Extrusion	Form cementum-like layer on the dentin surface	Cho et al. (2016)
Alveolar bone	GelMA	HERS^a^, DPSs^a^	Extrusion	Regenerate new bone *in vivo*	Tang et al. (2022)
Alveolar bone	GelMA, β-TCP	BMSCs	Extrusion	Enhance osteogenesis	Luo et al. (2022)
Alveolar bone	β-TCP/alginate OsteoInk™	Alveolarbone MSCs	Extrusion	Print scaffolds to fit the respective clinical defects	Anderson et al. (2022)
Alveolar bone	GelMA, PEGDA	PDLSCs	Inkjet	Regenerate new bone *in vivo*	Ma et al. (2017)
Alveolar bone	6% Mg-CS	—	DLP	Regenerate new bone *in vivo*	Qin et al. (2022)
Alveolar bone	silk fibroin, collagen, HA	rh-EPO	Extrusion	Regenerate new bone and collagen fibers *in vivo*	Liu et al. (2022a)
Alveolar bone	CSi-Mg10	—	Extrusion	Regenerate new bone *in vivo*	Shao et al. (2018)
Alveolar bone	Collagen, silk fibroin, nHA	KSL-W	Extrusion	Regenerate new bone *in vivo*	Li et al. (2022)
Alveolar bone	GelMA, PCL	DPSCs^a^	Extrusion	Promote OPN and OCN expression and CaP deposition	Buyuksungur et al. (2021)

^a^ Cells composing bioinks.

**TABLE 5:** Applications of dental composite tissue printing.

Tissue	Bioink	Cells/biological supplement	Technique	Results	Reference
Dentin-pulp complex	PCL, fibrinogen, gelatin, hyaluronic acid, glycerol	hDPSCs^a^	Extrusion	Regenerate patient-specific dentin-pulp complex *in vitro*	Han et al. (2019)
Dentin-pulp complex	PCL	hDPSCs/bioglass, hyaluronic acid	Extrusion	Regenerate dentin and dental pulp *in vitro*	Nejad et al. (2021)
Dentin-pulp complex	GelMA, dentin matrix molecules	—	DLP	Regenerate organized odontoblast layer and tertiary dentin	Cunha et al. (2023)
Periodontal complex	PCL with MgP	hMSCs	MEW	Regenerate PDL, bone and bone-ligament interfaces *in vivo*	Golafshan et al. (2023)
Periodontal complex	GelMA, dECM	hDFSCs^a^	DLP, DIW	Regenerate PDL, bone and bone-ligament interfaces *in vivo*	Yang et al. (2023)
Periodontal complex	PCL	—	MEW	Regenerate PDL and bone	Yao et al. (2022)
Periodontal complex	GelMA, sodium alginate	BMSCs^a^/BMP-2, PDGF	Extrusion	Regenerate bone and gingiva *in vivo*	Miao et al. (2023)
Periodontal complex	PCL, HA	DPSCs, PDLSCs, ABSCs/growth factors	Extrusion	Regenerate cementum, PDL and bone	Lee et al. (2014)
Periodontal complex	GelMA	DFSCs^a^	DLP	Functional periodontal regeneration *in vivo*	Ma et al. (2023)
Periodontal complex	PCL	Decellularized PDL cell sheet	MEW	Regenerate cementum, PDL and bone	Blaudez et al. (2023)
Periodontal complex	PCL	F/CaP coating	MEW	Regenerate cementum, PDL and bone *in vivo*	Daghrery et al. (2021)
Periodontal complex	PCL	hPDLSCs/F/CaP coating	MEW	Regenerate PDL and bone *in vivo*	Daghrery et al. (2023)
Periodontal complex	PCL	PDLSCs, fibroblasts	SLS	Regenerate PDL and bone *in vivo*	Pilipchuk et al. (2016)
Tooth root	PLA	DPSCs/HA coating	FDM	Higher degree of mineralization *in vivo*	Chen et al. (2021)
Whole tooth	PCL	SDF-1, BMP7	extrusion	Regenerate tooth-like structures *in vivo*	Kim et al. (2010)
Bio-root	TDM, PCL	DFSCs	extrusion	Regenerate anatomically shaped bio-root *in vivo*	Huang et al. (2023)
Bio-root	HA	DFSCs/nano-HA whiskers coating	DLP	Regenerate personalized bio-root with PDL-like enthesis formation	Chen et al. (2023)

^a^ Cells composing bioinks.

### 4.1 Soft tissue regeneration

#### 4.1.1 Dental pulp regeneration

Surrounded by rigid dentin walls, dental pulp is a loose connective tissue composed of cells (odontoblasts, fibroblasts, DPSCs), collagen, nerves and blood vessels. Retaining vital pulp is of great importance for tooth nutrient, repairment, and sensory, especially for immature permanent teeth. When dental pulp infected, root canal therapy (RCT) remains the first-option in clinical practice. However, tooth after RCT is faced with problems such as persistent inflammation, discoloration, and increased fragility (Ricketts, 2001). While pulp revascularisation presents a promising clinical approach, it fails to reconstruct functional pulp-like tissue and leads to unexpected calcification (Chen et al., 2012). Therefore, stem cell-based regenerative endodontic therapy has attracted the worldwide attention.

To achieve cellular pulp regeneration, it is essential to create a microenvironment conducive to stem cell proliferation and differentiation. Hydrogel proves to be a fitting candidate owing to its resemblance to the natural ECM. Various hydrogel have been evaluated for pulp bioprinting, encompassing fibrin, hyaluronic acid, GelMA and so on (Han et al., 2019; Nejad et al., 2021; Cunha et al., 2023).

Pulp vascularization is one of the key objectives as well as challenges in functional pulp regeneration. Several strategies have been utilized including the sacrificial material method and co-culture with endothelial cells (Athirasala et al., 2017). Duarte Campos et al. (2020) injected collagen based bioink into prepared root canal via a handheld bioprinter. The results of immunofluorescence showed successful vascularization in the root canal without the shrink of bioink. However, these strategies have yet to demonstrate their feasibility *in vivo*.

Remarkably, in a recently published work, Qian et al. successfully fabricated DPSC-loaded GelMA microspheres via a DLP printer. These cell-loaded microspheres have improved stemness and higher multi-directional differentiation potential, including angiogenic, neurogenic, and odontogenic differentiation. The regeneration of vascularized and neutralized pulp-like tissue was demonstrated by subcutaneous transplantation in nude mice and *in situ* experiment in swine (Qian et al., 2023).

#### 4.1.2 Periodontal ligament regeneration

Periodontal ligament (PDL) is a connective tissue that is mainly composed of collagen type I fibers and proteoglycans. Owing to the firm attachment between cementum and alveolar bone and specific oriented collagen fibers, it provides mechanical stability and attenuate the masticatory stress to protect tooth. Furthermore, PDL contains an abundance of blood vessels and nerve endings that provide nourishment to the cementum and alveolar bone. Utilizing mesenchymal stem cells present in the periodontium, repairment and remodeling of periodontal tissue are possible (Hurng et al., 2011). Hence, the PDL regeneration is an essential part of periodontal regeneration.

3D printing holds its intrinsic advantage to form highly-arranged collagen fibers because of the exist of micro-strands and precise control of cell position in the 3D network. It has been proven that compared with cell-seeding bulk collagen, the cell-laden collagen by bioprinting showed significantly more lang periodontal ligament-like soft tissue on the surface of titanium implants (Lee U.-L. et al., 2021).

To physically control the orientations of fiber bundles, so called “fiber-guiding scaffold,” has emerged and attracted increasing focus. Lin H.-H. et al. (2021) fabricated a collagen-based waveform microfibers and evaluated the microcrimped scaffold under shear load (6 dyne/cm^2^). Compared with straight microfibers, waveform microfibers exhibited enhanced cell spreading and adhesion, as well as upregulated expression of periostin (a key molecule for extracellular matrix assembly).

Kim and Park (2020) created microgroove patterns on the scaffold surfaces with different slice intervals. After 7 days culture, the μG-25 (25.40 µm intervals) showed the ability to organize aligned PDL cells while the μG-6 (6.35 µm intervals) leaded random organization. Similarly, Pilipchuk et al. (2016) employed 3D-printed scaffold combined with micropatterned film to achieve regeneration and integration of alveolar bone and PDL. Their conclusions support that optimized surface tomography induce specific orientations of PDL fibers in a more predictable manner.

#### 4.1.3 Gingival regeneration

Gingiva is another key component of oral soft tissue, which is essential for oral structural integrity and dental aesthetics. Although autogenous gingival grafts have been widely used to reverse gingival recession and augment periodontal soft tissue, the lack of donor tissue sources, the complexity of the surgical procedures, and the postoperative pain and numbness still hamper a good prognosis (Yang M. et al., 2019). Therefore, the tissue engineering strategy seems to be a new option for regenerative oral soft tissue augmentation. Tang along with his team developed a kind of bioink composed of alginate, gelatin and injectable platelet-rich fibrin (i-PRF), which can release various repair-related growth factors. *In vivo* experiments, 3D-printed constructs with 50% i-PRF showed more collagen formation and host tissue infiltration compared to the group without. In their latest study, they bioprinted acellular dermal matrix (ADM)-based hydrogel composite and performed *in vivo* implantation in beagles, demonstrating that GF-loaded ADM scaffolds significantly promote keratinised gingival regeneration (Yi et al., 2022; Liu et al., 2023).

### 4.2 Hard tissue regeneration

#### 4.2.1 Enamel regeneration

Dental enamel is the hardest tissue in the human body, providing external protection for the tooth against masticatory forces. As a kind of non-vascular, acellular, highly mineralized hard tissue, enamel is composed of substituted hydroxyapatite and organic macromolecules (Nanci, 2013). Since the enamel organ disappears at the end of the tooth germ development, there are no enamel epithelial cells in a mature tooth, resulting in the lack of enamel regeneration ability. Thus, development of alternative approaches to repair and regenerate enamel defects is much needed but remains challenging due to the highly-ordered hydroxyapatite structure. Zhao et al. (2022) used supergravity preparation technology and hydrothermal treatment to disperse the Hap nanorods in the hybrid resin-based composites (RBCs), developing a new bioactive ink. The printed tooth crown possesses multi-scale highly ordered structure and strong mechanical strength. Mohabatpour et al. (2022) combined carboxymethylcellulose (CMC) and alginate to create a new bioink that induced the differentiation of dental epithelial cells (HAT-7) to enamel-forming cells and promoted Ca and P deposition as well as matrix mineralization *in vitro*.

#### 4.2.2 Dentin regeneration

Human dentin is composed of hydroxyapatite (70% by weight) and an organic matrix (35% by weight) of collagenous and noncollagenous proteins, such as dentin sialophosphoprotein (DSPP), dentin matrix protein-1 (DMP-1), osteopontin, and osteocalcin (Zhang et al., 2014). Dentin regeneration is one of the most explored parts in the field of dental tissue engineering. 3D printing is capable of forming customed macro structures and intricated interconnections. Compared with conventional cast-molded hydrogel, 3D-printed alginate/gelatin scaffold can better promote the cell adhesion, viability, and osteogenic/odontoblastic differentiation of hDPSCs (Yu et al., 2019). Besides, 3D printed Biodentine/PCL scaffold exhibited a good apatite-forming ability and induced hDPCs proliferation and differentiation (Ho et al., 2018).

Incorporation of biological cues also helps to induce the odontogenic differentiation of stem/progenitor cells, and then promote the secreting and mineralization of dentin matrix. The extracts of dentin matrix, both soluble and insoluble molecules, significantly enhanced odontogenic differentiation of SCAPs encapsulated in bioprinted hydrogels (Athirasala et al., 2018). Similarly, Han et al. (2021) mixed DDM particles with fibrinogen-gelatin to produce a DDM particle-based bio-ink, which enhanced the dentin-oriented differentiation of DPSCs. Besides, the study by Kim et al. indicated that supplemented with bone-derived dECM can accelerate the odontogenic differentiation of hDPSCs (Kim et al., 2022).

In addition to bioactive molecules, Yeh et al. (2022) fabricated a mesoporous calcium silicate/calcium sulfate (MSCS) scaffold and added quercetin, a novel bioactive molecule which can reduce inflammatory mediator and inhibit osteoclast activity, into the printed scaffold. The composite scaffold not only possessed better physicochemical and biological properties but also enhanced odontogenic and immuno-suppressive properties. Feng et al. (2021) coated a 5 nm thick titania layer via atomic layer deposition on the 3D printed PLA scaffold, and higher DPSCs plating efficiency and upregulated expression of osteocalcin compared with uncoated PLA scaffolds were observed.

#### 4.2.3 Alveolar bone regeneration

Alveolar bone loss is often caused by periodontitis or peri-implant inflammation. Regeneration of alveolar bone is one of the goals of periodontal therapy, and it is also a prerequisite for implant restoration. Compared with other craniomaxillofacial bone constructs, alveolar bone scaffold design and manufacture as a tooth-supporting tissue remains a challenge due to the structural complexity and geometric adaptability of tooth-root surface.

Bioceramics play a crucial role in the bone tissue engineering. Qin et al. explored the effect of different pore dimensions (Ø 480, 600, and 720 μm) on mechanical properties, bioactive ion release, and bio-dissolution of bioceramic scaffolds. The 600 μm group exhibited maximal new bone formation in the rabbits’ mandibular bone defects model (Qin et al., 2022). In another study, Osteoink™, composed of hydroxyapatite and α-TCP, was utilized to develop calcium phosphate (CaP)-based bioceramic scaffolds for alveolar bone reconstruction. The printed scaffolds had a high degree of accuracy to fit the respective clinical defects for which they were designed (Anderson et al., 2022). A newly developed bioceramic material, ∼10% Mg-substituted wollastonite (Ca90%Mg10%SiO3; CSi-Mg10) was also used to fabricated customed scaffolds for reconstruction of alveolar bone defect, exhibited desired osteoconduction, osteoinductivity and adequate biodegradation (Shao et al., 2018).

GelMA is another well-established material for alveolar bone reconstruction by virtue of its analogue to extracellular matrix and adjustable physicochemical properties. 3D-printed PCL/GelMA hybrid scaffolds yielded cell viability, osteogenic differentiation of DPSCs and mineralization *in vitro* (Buyuksungur et al., 2021). Tang et al. (2022) combined HERS cells and dental papilla cells (DPCs) to mimic the micro-environment of cell-cell interaction. Plenty of mineralization texture were observed when transplanted into the rat’s alveolar sockets. GelMA/PEGDA hybrid hydrogel were fabricated by controlling the volume ratio of GelMA-to-PEGDA. The results indicated that the 4/1 GelMA/PEGDA composite hydrogel exhibited best performance in osteogenic differentiation and formed new bone tissue *in vivo* (Ma et al., 2017). Luo et al. (2022) developed GelMA/β-TCP hybrid scaffolds which showed good biocompatibility and mechanical properties, and stimulated osteogenesis of BMSCs.

Furthermore, the supplement of growth factors, peptides, and other bioactive substances undoubtedly endows 3D printed constructs with superior biological properties, such as antimicrobial peptide KSL-W (Li et al., 2022), recombinant human erythropoietin (rh-EPO) (Liu H. et al., 2022) and graphene oxide (Park et al., 2021).

### 4.3 Multi-tissue regeneration

#### 4.3.1 Dentin-pulp complex regeneration

The dentin and dental pulp derived from dental papilla, which is formed during tooth germ development and participate in tooth activities together as the dentin–pulp complex (Abbass et al., 2020). Dentin offers durable shielding for the pulp, whereas the pulp supplies essential nutrients for the dentin and generates tertiary dentin as a response to caries, mechanical injury, or acid erosion. Therefore, the regeneration of vital dentin-pulp complex is the ideal target for endodontic medical treatment.

It has been widely studied that the fate of stem cells is closely associated with ECM characteristics (Liu et al., 2018). In this regard, Han et al. (2019) developed hDPSCs loaded bioink with different fibrinogen content to print dentin and pulp tissues (F20 for dentin, F5 for dental pulp) respectively. Bilayer scaffolds with region-specific induced soft and hard tissues are likewise seen as one of the viable strategies. Nejad et al. (2021) constructed PCL/45S5 bioglass (BG)-PCL/hyaluronic acid (HyA) scaffold and evaluated the physicochemical properties of the hybrid scaffold. The results showed that PCL/BG scaffolds had superior mechanical strength and surface roughness, and the PCL/HyA scaffolds possessed increased hydrophilicity for cell adhesion. Recently, Cunha et al. (2023) utilized 3D-printed microgels doped with dentin matrix molecules for direct dental pulp capping. Although both the microgel group supplemented with DMM particles and the MTA group showed the formation of organized pulp tissue, dentin-like tissue and new blood vessels, the presence of DMM helped to promote dentin bridge formation and exhibited more tertiary dentin deposition and attenuate less pulp necrosis.

#### 4.3.2 Periodontal complex regeneration

Periodontitis is one of the most prevalent oral diseases in the world, resulting in periodontal attachment loss, alveolar bone resorption, even tooth loss (Kwon et al., 2021). The goal of periodontal treatment is to control the infection and reconstruct the functional tooth-supporting tissue. Guided tissue regeneration (GTR) is currently gold standard of periodontal tissue reestablishment by a barrier membrane to allow selective repopulation of PDL cells and bone, yet it still suffers from various limitations as well as clinical variability (Karring et al., 1993). The periodontal complex comprises multiple tissues, including periodontal ligament, cementum and alveolar bone, forming a “sandwich-like” structure. Herein, multiphasic scaffolds to mimic the cementum-PDL-alveolar bone complex has increasingly attracted the attention of researchers (Ivanovski et al., 2014).

In previous studies, Lee CH and his colleagues developed various growth factor-releasing scaffolds by loaded PLGA microspheres for periodontal tissue regeneration. They initially achieved integrated cementum-like tissue on the surface of dentin with high expression of cementum matrix protein 1 (CEMP1), an important marker of cementogenesis (Cho et al., 2016). Their another study used multiphase scaffolds for periodontium complex regeneration via spatiotemporal delivery of multiple bioactive cues, involving recombinant human amelogenin, CTGF and BMP-2. They also demonstrated the different microstrands spacing have an effect on the generation of integrated multiple tissues (Lee et al., 2014). Consistent with Lee’s studies, Daghrery et al. engineered PCL scaffold with F/Ca coating and revealed that aligned fibers strongly support ligamentogenesis and scaffold architecture with 500 μm strands spacing supports osteogenesis. The results of Masson’s trichrome staining indicated that the tissue-specific scaffold induced the regeneration of both soft and hard tissue *in vivo* (Daghrery et al., 2021; Daghrery et al., 2023). Furthermore, a novel composite hydrogel was designed as cell-laden bio-ink, encapsulating BMP-2 and PDGF that effectively facilitate alveolar bone regeneration and promote gingival healing respectively in the periodontal defect model of beagles (Miao et al., 2023). Ma et al. (2023) reported biomimetic periodontium patches (BPPs) by DLP technology. The BPPs featuring correctly oriented fibers and the assessment of clinically functional properties proved the regenerative periodontium can stand orthodontic migration force to achieve stable tooth movement.

Another important challenge to be solved is the interface integration. Soft-to-hard tissue interfaces have always been a tricky problem in tissue engineering for the difficulty to reproduce interface enthesis (Calejo et al., 2019). To overcome the lack of adhesion between newly formed PDL and bone, Blaudez et al. (2023) designed a decellularized construct consisting of consisting of PDL cell sheet and PCL scaffold for tissue-specific periodontal regeneration. Before decellularization, the biphasic construct was cultured for 24 h to allow cell sheet adhesion and enhance the cohesion between periodontium and bone part. Yao et al. (2022) introduced a transition region with 500-µm filament spacing, while the bone region was 250-μm filament spacing and the PDL region was consisted of aligned PCL filaments. The transition region separated the tissue-specific regeneration in the other two parts at the early time and promoted cellular cross-communication later between two regions. The insertion of ligamentous fibers towards the bone and calcium gradient were observed in the transition space. Similarly, Golafshan et al. (2023) fabricated a tri-layer scaffold in which PCL matrix with MgP was for bone, highly aligned PCL fibers were presented for PDL and random PCL fibers were printed to mimic the bone-to-PDL interface. The tri-layer scaffold showed enhanced mechanical stability and achieved coordinated periodontal tissue regeneration in the rodent model. Moreover, a recent study reported a biomimetic periodontal module with high architectural integrity. The module provided high-precision topographical cues and biochemical environment conducive to regulating cell behavior and achieving periodontal regeneration with the enthesis of the bone-ligament interface and well-aligned fibers in a beagle model (Yang et al., 2023).

#### 4.3.3 Whole tooth/bio-root regeneration

Whole tooth regeneration with natural tooth morphology and function is the ultimate goal of dental regenerative medicine. Back in 2010, Kim et al. fabricated anatomically shaped human molar scaffolds and rat mandibular incisor scaffolds by 3D printing and performed orthotopic implantation of rat incisor scaffolds, combined with the delivery of SDF1 and BMP7. After 9 weeks, the histological results revealed regeneration of the PDL-like tissue and new bone at the interface between the scaffolds and native alveolar bone (Kim et al., 2010). While it is possible to replicate the morphology of natural teeth, grafts often struggle to form a strong enough periodontal bond with the host jawbone in a short period of time, which can compromise their ability to withstand substantial occlusal forces.

Tooth germ recombination appears to be another viable path to achieving whole tooth regeneration (Zhang and Yelick, 2021). Studies have been conducted to generate bioengineered tooth germs by the reconstruction of dental epithelial and mesenchymal cells (Ono et al., 2017). However, it is still tough to regenerate eruption-competent tooth germs hence the epithelial-mesenchymal transition during tooth development is yet to be completely unveiled and replicating spatiotemporal interactions between different cells remains challenging. 3D bioprinting technology is thought to offer new possibilities for bioengineered tooth germ due to its ability to precisely control microstructures at the cellular level.

Therefore, fabricating bioengineered roots, also known as “bio-root,” and then restoring with artificial crown seem to be a much more feasible approach. Bio-root, referred to the bioengineered tooth root, was firstly proposed by Sonoyama in 2006 and has demonstrated the ability to regenerate dentinal tubule-like and periodontal ligament-like structures in swine model (Wei et al., 2013). Up to recently, Huang et al. developed a personalized 3D-printed scaffold with PCL and TDM. Notably, the TDM scaffolds combined with DFSC sheets served as an analogue to natural tooth root and with satisfied angiogenic capacity in orthotopic transplantation in beagles (Huang et al., 2023).

Besides, a study by Chen et al. fabricated personalized bio-root by DLP printing of hydroxyapatite bioceramic. Bioceramic sintered at 1,250°C exhibited almost twice elastic modulus than natural decellularized dentine, which significantly enhanced the physicochemical properties of Hap. The nano-HAw (nano-hydroxyapatite whiskers) coating improved both mechanical property and surface hydrophilicity, and periodontal ligament-like enthesis formation in-situ transplantation in rat alveolar fossa (Chen et al., 2023).

## 5 Discussion

Although restorative treatment is still the mainstream treatment, regenerative treatment is gradually occupying a place in dentistry clinical practice. Notably, 3D printing technology is a promising solution in the field of regenerative medicine. 3D printing holds inherent advantages of construct geometric topologies that affect cell behavior and reproduce intricated microstructure to mimic the natural tissue/organ physiochemical and biological properties.

In general, the materials used for 3D printing-based tooth regeneration should conform the following characteristics: 1) bioactivity with stem/progenitor cells; 2) ease of processing; 3) proper mechanical properties that match the corresponding tissue; 4) ideal physical characteristics, such like porosity, surface roughness, wettability, and viscosity. The types of biomaterials commonly used in 3D dental printing are summarized above. Currently, composite materials are gaining increasingly focus for the single material fail to yield heterogeneous constructs. Besides, biological agents are integrated to the printed constructs for multifunctional biological effects, such like growth factors, mRNA, exosomes, and drugs (Zhou et al., 2018).

Dentoalveolar tissue exhibits compartmentalized architectures with structural integrations. Though studies referred to in this review has achieved regeneration of dental tissues to varying degrees, there are challenges need to be addressed, such as adequate vascularization, neuralization, and internal integration into the host tissue. Moreover, for the acellular hard tissues subjected to strong mechanical loads, the balance between prolonged mineralization and scaffold degradation is still a tackle problem. The regeneration of a single tissue cannot meet the needs of functional tooth reconstruction, herein, multi-tissue biofabrication of dental tissue has emerged as a prominent area of investigation. The utilization of multiphasic scaffolds with designed tissue-specific organization for detin-pulp and periodontal complex have been discussed above. However, scaffolds fabricated in this way usually lack interphasic cohesion and lead to compromised stability of printed constructs.

To better mimic natural tooth tissues and achieve functional tooth reconstruction, further research should be undertaken in several areas. One is to develop novel 3D printing technique with higher printing resolution. Up to now, extrusion-based 3D printing is the most used printing technique in the field of tissue engineering by virtue of easy access to material and low cost, but the printing precision is not comparable to laser-assisted printing. Secondly, the regulation of stem cell fate by the biomechanical properties of the extracellular matrix needs to be further explored, which will inspire the cell-matrix interaction in the printing architecture. The emerging 4D printing is capable of creating scaffolds that can create controlled dynamic cellular microenvironment, providing a potential tool for recapturing the natural process of tooth genesis and restoration (Wang et al., 2022). Another direction of development lies in immunomodulation for 3D printed constructs. The biomaterial scaffolds often trigger the host immune rejection *in vivo*, leading to the chronic inflammation and eventually regeneration failure (Whitaker et al., 2021). Only a few studies have reported the immune effects of 3D printed dental substitutes, including modulation of inflammatory factors and macrophage polarization.

Whilst the laboratory studies about 3D printing for dental tissue are encouraging, they are yet to be translated into clinical practice. There have only been a limited number of studies which have reported on the clinical evaluation of 3D-printed constructs in the human body. To bridge the gap between laboratory and clinical applications, further safety and function tests are urgently required. In conclusion, 3D printing technology displays promising translational and therapeutic potential for the regeneration of dental tissue, which lays a strong foundation for regenerative medicine.
